# Aquaporin-1 Deficiency Protects Against Myocardial Infarction by Reducing Both Edema and Apoptosis in Mice

**DOI:** 10.1038/srep13807

**Published:** 2015-09-08

**Authors:** Lihua Li, Zhiyong Weng, Chenjuan Yao, Yuanlin Song, Tonghui Ma

**Affiliations:** 1 Liaoning Medical University, Department of Cell Biology, Jinzhou, PR China; 2 The University of Tokushima Graduate School, Department of Molecular Oral Physiology, Tokushima, Japan; 3 Fudan University, Department of Pulmonary Medicine, Shanghai, PR China; 4 Dalian Medical University, Department of Physiology, Dalian, PR China

## Abstract

Many studies have determined that AQP1 plays an important role in edema formation and resolution in various tissues via water transport across the cell membrane. The aim of this research was to determine both if and how AQP1 is associated with cardiac ischemic injury, particularly the development of edema following myocardial infarction (MI). AQP1^+/+^ and AQP1^−/−^ mice were used to create the MI model. Under physiological conditions, AQP1^−/−^ mice develop normally; however, in the setting of MI, they exhibit cardioprotective properties, as shown by reduced cardiac infarct size determined via NBT staining, improved cardiac function determined via left ventricular catheter measurements, decreased AQP1-dependent myocardial edema determined via water content assays, and decreased apoptosis determined via TUNEL analysis. Cardiac ischemia caused by hypoxia secondary to AQP1 deficiency stabilized the expression of HIF-1α in endothelial cells and subsequently decreased microvascular permeability, resulting in the development of edema. The AQP1-dependent myocardial edema and apoptosis contributed to the development of MI. AQP1 deficiency protected cardiac function from ischemic injury following MI. Furthermore, AQP1 deficiency reduced microvascular permeability via the stabilization of HIF-1α levels in endothelial cells and decreased cellular apoptosis following MI.

Myocardial infarction (MI) may result in myocardial edema, which is directly associated with mortality due to impairment in both left ventricular systolic and diastolic function^1^,^2^,^3^. Myocardial edema occurs primarily as a result of irreversible myocardium injury secondary to myocyte swelling, which results in cardiac dysfunction^4^,^5^. Increased myocardial microvascular filtration rates and decreased myocardial lymph flow rates are two major factors associated with the development of interstitial myocardial edema following MI^6^. However, increased microvascular permeability does not necessarily cause myocardial edema^7^. Therefore, another mechanism may be associated with this process. However, data are limited concerning the molecular mechanisms underlying the development of myocardial edema following MI.

Aquaporins (AQPs) are water-transporting membrane proteins selectively expressed in the cells of various organs, wherein they perform important physiological functions^3^,^4^,^8^,^9^,^10^,^11^. Several studies involving AQP1 knockout mice have demonstrated that AQP1 is expressed in the microvasculature and the endothelium of cardiac tissue as determined via Western blotting and RT-PCR. AQP1 also facilitates osmotic water transport in cardiac membrane vesicles, although previous immunostaining studies have been unable to confirm its presence in cardiac myocytes^12^. AQP4 has been detected within mouse hearts at the protein level^13^ and has a water transport capacity as much as 24 times that of AQP1; however, AQP4 has not been shown to increase the water permeability of cardiac membrane vesicles^14^. Therefore, AQP4 is considered physiologically irrelevant in the mouse heart^15^. Recent studies have implicated AQP1 as a mediator of cardiac damage in the setting of both myocardial ischemia and edema.

The significance of cardiac AQP1 expression and its related functions remain unclear. Therefore, we investigated the role of AQP1 following MI by comparing heart morphology, infarct size, myocardial water content, cardiac function and hypoxia-inducible factor-1α (HIF-1α) levels and cellular apoptosis between AQP1^−/−^ and AQP1^+/+^ mice. We observed that AQP1 deficiency significantly decreased myocardial infarct size, and also markedly reduced cardiac edema, stabilized HIF-1α levels and decreased both microvascular permeability and cellular apoptosis following MI, which may have been responsible for the improvements in the cardiac function of the AQP1 deficient mice.

## Results

### Cardiac non-changes due to AQP1 deficiency

**Figure 1:**
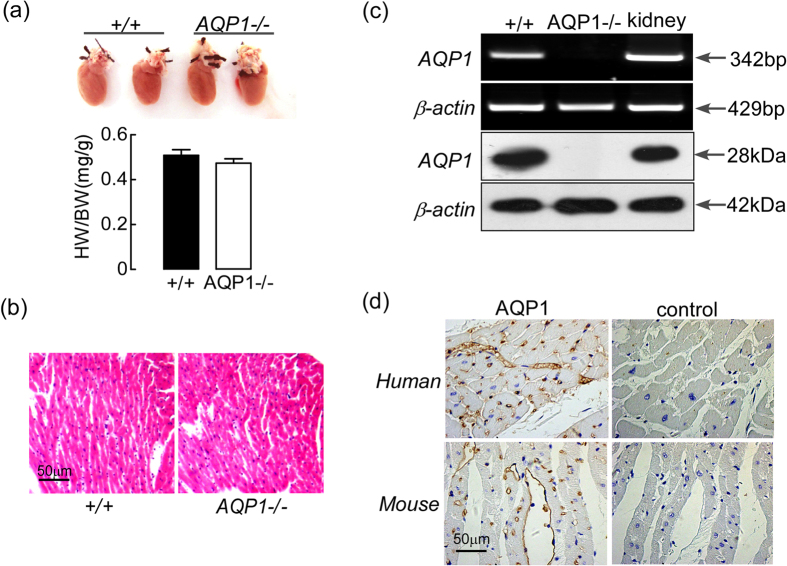
Normal cardiac morphology and the expression of AQP1 in AQP1^−/−^ and AQP1^+/+^ mice. ((**a**) top) Heart images from the AQP1^−/−^ and AQP1^+/+^ mice. ((**a**) bottom) The heart weight/body weight ratio was representative of adult mice at 8–10 weeks age. The values are expressed as the mean ± SEM. (n = 6, differences not significant). (**b**) Heart sections from the AQP1^−/−^ and AQP1^+/+^ mice stained with H&E. Scale bar: 50 μm. (**c**) The RT-PCR (top) and Western blot (bottom) analyses demonstrate the relative expression levels of AQP1 in the AQP1^−/−^ and AQP1^+/+^ mice hearts. Kidney tissue was used as positive control. (**d**) The AQP1 expression pattern in the normal hearts of both humans (left) and mice (right) as demonstrated via immunohistochemistry. Scale bar: 50 μm.

Figure 1a, *top,* depicts the normal hearts of the AQP1^+/+^ and AQP1^−/−^ mice. The hearts of the AQP1^+/+^ and AQP1^−/−^ mice exhibited similar sizes, gross anatomical features and weights [Fig. 1(a), bottom]. H&E staining demonstrated that the hearts of the AQP1^+/+^ and AQP1^−/−^ mice exhibited similar histological features, as well as comparable myocardium thicknesses and myocyte densities [Fig. 1(b)]. AQP1 immunohistochemistry staining demonstrated that the endothelial cells exhibited expression patterns consistent with those of normal human hearts and AQP1^+/+^ mouse hearts [Fig. 1(d)], as brown staining was visible across the membranes of the endothelial cells and limited staining of the myocytes was visible. No specific staining was observed in the hearts of the control slices. The expression of AQP1 in human heart exhibits a pattern similar to that observed in the mouse heart.

Figure 1(c), top, depicts RT-PCR with respect to mouse AQP1; cardiac RNA expression in both the AQP1^+/+^ and the AQP1^−/−^ mice was used as template. The expression of the transcript encoding AQP1 was observed in the hearts of the wild type mice using cDNA. The Western blot analysis of the expression level of AQP1 protein in both the AQP1^+/+^ and the AQP1^−/−^ mice is depicted in Fig. 1(c), bottom. Strong expression bands of AQP1 mRNA and protein were observed in the hearts of the AQP1^*+/+*^ mice. Kidney tissue was used as a positive control.

### AQP1 deficiency reduced myocardial infarct size following MI

**Figure 2:**
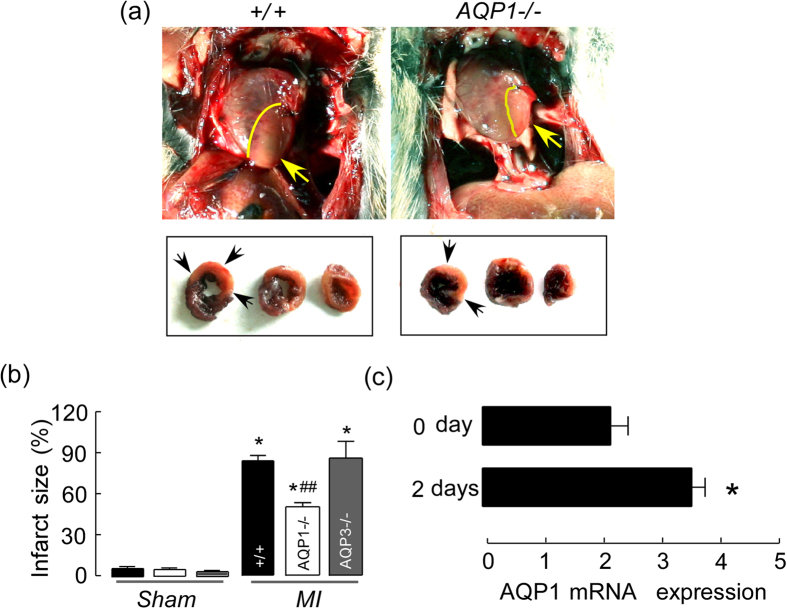
AQP1 deficiency reduced myocardial infarct size at 2 days following MI. ((**a**) top) depicts representative images of the heart *in situ*, with ligation of the left anterior descending coronary artery. ((**a**) bottom) depicts representative samples of heart slices taken at the mid-papillary muscle level following MI. The perfused myocardium was stained using Evans blue solution, whereas the occluded vasculature remains uncolored. The arrow indicates the ischemic site. (**b**) The infarct size is expressed as the percentage of the area at risk, AR. The data are expressed as the mean ± SEM (n = 15, *P < 0.001 *vs* sham. ^##^*P* < 0.01 *vs* AQP1^+/+^ and AQP3^−/−^ MI mice). (**c**) Quantitative real-time RT-PCR demonstrating AQP1 expression in cardiac tissue following MI 0 day and day 2. The values are expressed as the mean ± SEM (n = 6, *P < 0.01 *vs*. day 0).

In order to assess the severity of cardiac injury following MI, cardiac infarct size was measured via NBT staining on day 2 following MI in the AQP1^+/+^ and AQP1^−/−^ mice [Fig. 2(a)]. Compared with the AQP1^+/+^ mice, the AQP1^−/−^ mice exhibited significantly smaller infarct size [Fig. 2(b)]. Small infarct sizes were also observed in the control group (sham operated mice) compared with the AQP1^+/+^ and AQP1^−/−^ mice [Fig. 2(b)]. In order to determine if infarct size reduction was a generalized phenomenon in the AQP deficient mice, we also induced MI in AQP3^−/−^ mice and measured myocardial infarct sizes. No differences were observed between the AQP3^+/+^ and AQP3^−/−^ mice; however, myocardial infarct size was markedly reduced in the AQP1^−/−^ mice compared with the AQP3^−/−^ mice [Fig. 2(b)]. Figure 2(c) depicts the expression of AQP1 following MI, as said expression was up-regulated compared with before MI. These findings suggest that compensatory upregulation of the other AQPs exerted protective effects in the AQP1^−/−^ mice. We also analyzed the expression of the other AQPs following MI via RT- PCR. However, none of the other AQPs expression levels was up-regulated in the AQP1^−/−^ mice (data not shown). These results are indicative of the involvement of AQP1 in both the pathophysiology and the impairments that occur following MI.

### AQP1 deficiency protected cardiac contractile function following MI

**Figure 3:**
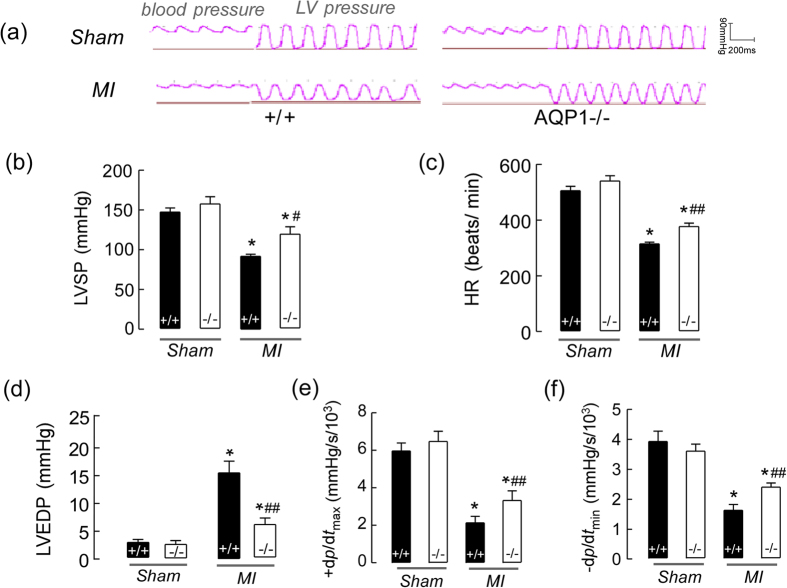
The effects of AQP1 deficiency on cardiac contractile function following MI. (**a**) A representative tracing of LV hemodynamic recordings obtained *in vivo* using a conductance catheter in both the sham-operated mice and both the AQP1^−/−^ and AQP1^+/+^ mice at 18 hours following MI. (**b**) The LV systolic pressures (LVSPs) (n = 12), heart rates (HRs) (n = 12) (**c**), LV end-diastolic pressures (LVEDPs) (n = 12) (**d**) and maximal positive (**e**) and minimal negative (**f**) first derivatives of LV pressure (+d*P/*d*t*_max_ and –d*P*/d*t*_min_) (n = 12). The values are expressed as the mean ± SEM. *P < 0.01 *vs* sham; ^#^*P* < 0.05, ^##^*P* < 0.01 *vs* AQP1^+/+^ MI mice.

In order to determine the role of AQP1 in cardiac function following MI, we assessed HR, LVSP, LVEDP, +d*P*/d*t*_max_ and −d*P*/d*t*_min_ *in vivo* at 18 hours following MI using an LV catheter that simultaneously measures pressure, as depicted in Fig. 3(a). LVSP (b), HR (c), and +d*P*/d*t*_max_ (e), as well as −d*P*/d*t*_min_ (f), were significantly decreased in the AQP1^+/+^ and AQP1^−/−^ MI mice, whereas LVEDP (d) was increased compared with the control mice. However, LV +d*P*/d*t*_max_ and −d*P*/d*t*_min_ were greater, and LVEDP was smaller in the AQP1^−/−^ mice compared with the AQP1^+/+^ mice. These results suggest that AQP1 deficiency protected against cardiac functional impairment following MI.

### AQP1 deficiency attenuated myocardial edema following MI

**Figure 4:**
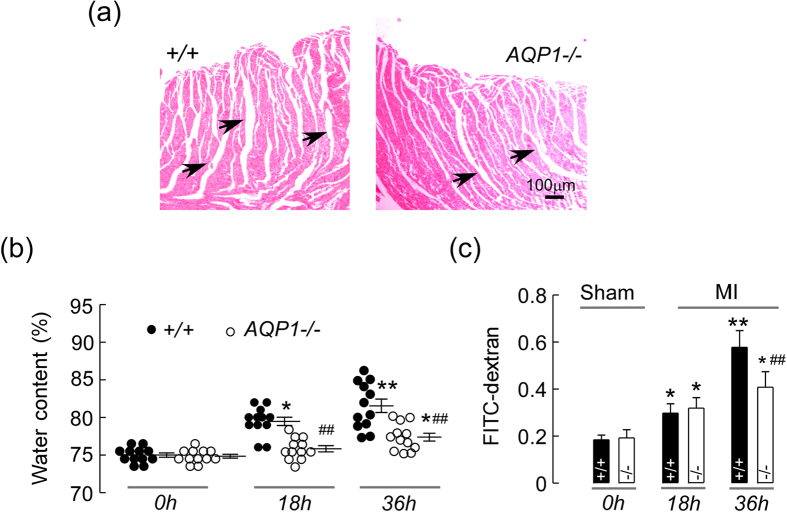
The effects of AQP1 deficiency on myocardial edema following MI. (**a**) Myocardial interstitial edema was observed via H&E staining in the AQP1^+/+^ (left) and AQP1^−/−^ mice (right) at 18 h following MI. Scale bar: 100 μm. (**b**) A quantitative analysis of myocardial water content at 0 h, 18 h and 36 h following MI in the AQP1^−/−^ and AQP1^+/+^ mice using both dry and wet weights. The values are expressed as the mean ± SEM (n = 12, *P < 0.05, **P < 0.01 *vs* 0 h. ^##^*P* < 0.01 *vs* AQP1^+/+^ mice at 18 h and 36 h). (**c**) A quantitative analysis of microvascular permeability via measurements of FITC-dextran leakage using spectrophotometry (n = 6, *P < 0.05, **P < 0.01 *vs* 0 h. ^##^*P* < 0.01 *vs* AQP1^+/+^ mice at 36 h).

AQP-facilitated plasma membrane water permeability reportedly promotes the development of edema in a variety of tissues^8^. In order to determine whether this mechanism is applicable to cardiac tissue, we assessed the development of AQP1-dependent myocardial edema following MI via the following methods: a myocardial water content assay and microvascular permeability assay. We first compared myocardial water content in the AQP1^−/−^ and AQP1^+/+^ mice at both 18 and 36 h following MI. As demonstrated in Fig. 4(a), MI caused in increase in the water content of the myocardium interstitial space, as determined via H&E staining. The AQP1^−/−^ mice exhibited significant smaller myocardial interstitial spaces compared with the AQP1^+/+^ mice. Figure 4(b) also depicted similar reductions in the water content of the AQP1^−/−^ MI mice using both the wet and dry weights of the infarct myocardium. The edema peaked at 36 h following MI in the AQP1^+/+^ mice. The microvascular permeability assay exhibited no significant differences between the AQP1^−/−^ and AQP1^+/+^ mice secondary to FITC-dextran leakage, at 18 h following MI, as demonstrated in Fig. 4(c). These results support the involvement of AQP1 in the development of myocardial edema following MI. However, at 36 h following MI, FITC-dextran leakage was significantly increased in the AQP1^+/+^ MI mice compared with the AQP1^−/−^ MI mice. As AQP1^−/−^ mice suffer water loss under stress due to deficient kidney re-absorption, we used AQP3^−/−^ mice in order to avoid water loss. However, no differences were observed in myocardial water content between the AQP3^+/+^ and AQP3^−/−^ mice (data not shown). These results indicated that increased microvascular permeability exacerbated AQP1-dependent myocardial edema.

### AQP1 deficiency stabilized the expression of HIF-1α following MI

**Figure 5:**
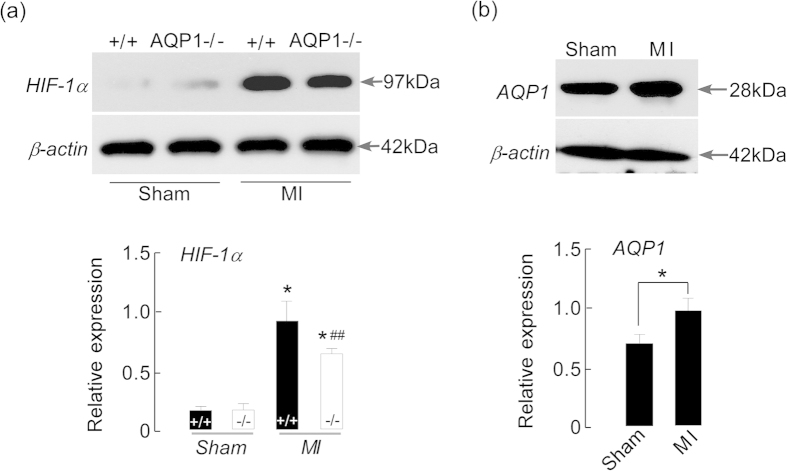
The effects of AQP1 deficiency on the expression of HIF-1α and AQP1 at 24 hours after MI. (**a**) Photographs and a quantitative analysis of the expression of HIF-1α via Western blotting in the AQP1^−/−^ and AQP1^+/+^ mice. The HIF-1α expression level was significantly increased following MI in the AQP1^+/+^ mice compared with the AQP1^−/−^ mice (n = 6, *P < 0.01 *vs* sham. ^##^*P* < 0.01 *vs* AQP1^+/+^ MI mice). (**b**) Photographs and a quantitative analysis of the expression of AQP1 via Western blotting in the AQP1^+/+^ MI mice compared with the sham-operated AQP1^+/+^ mice (n = 6, *P < 0.01).

In order to determine the cellular mechanisms underlying AQP1-dependent myocardial edema, we examined HIF-1α levels in cardiac tissue following MI. Increases in the HIF-1α levels of vascular endothelial cells represent one of the earliest responses to myocardial ischemia and infarction^16^. Our previous *in vitro* studies have demonstrated that AQPs facilitate the secretion of specific cytokines^17^. Therefore, the HIF-1α level during the early phase of MI was assayed via immunoblotting. Figure 5(a) demonstrates that the expression of HIF-1α in the AQP1^+/+^ MI mice was significantly increased compared with the AQP1^−/−^ MI mice. Moreover, the level of AQP1 expression in the MI mice was also increased compared with the control mice [Fig. 5(b)]. The level of HIF-1α expression correlated with AQP1 expression and reinforced its involvement in MI.

### AQP1 deficiency inhibited cardiac apoptosis following MI

**Figure 6:**
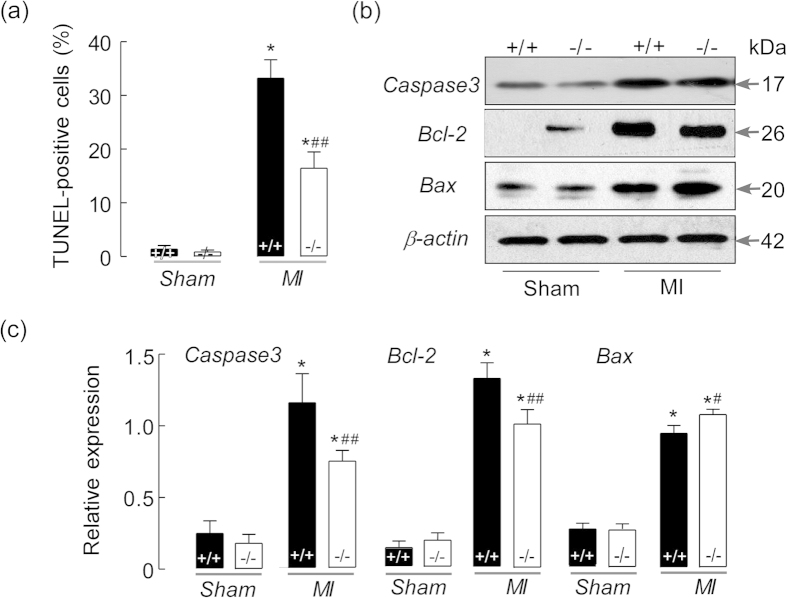
The effects of AQP1 deficiency on cardiomyocyte apoptosis following MI. (**a**) A quantitative analysis of apoptotic cells in the hearts of mice via a TUNEL assay (n = 6, *P < 0.001 *vs* sham. ^##^*P* < 0.01 *vs* AQP1^+/+^ MI mice). TUNEL quantification was based on 6 fields per heart and 6 hearts per group. (**b**) The heart tissues from the AQP1^+/+^ and AQP1^−/−^ mice at 48 h following MI were lysed and subjected to Western blotting in order to analyze the expression of caspase-3, bcl-2 and bax. (**c**) Band densities were measured using Image J software and normalized to β-actin. The data are expressed as the mean ± SEM (n = 6, *P < 0.01 *vs* sham; ^#^*P* < 0.05, ^##^*P* < 0.01 *vs* AQP1^+/+^ MI mice).

In order to determine whether AQP1-dependent myocardial edema mediates cardiac myocyte injury, an apoptosis analysis was undertaken using terminal deoxynucleotidyl transferase dUTP nick-end labeling (TUNEL) staining, and revealed that in non-ischemic heart tissue, the number of apoptotic cells were similar in both the AQP1^+/+^ MI mice and the AQP1^−/−^ MI mice. However, the number of apoptotic cells in the ischemic heart tissue of the AQP1^+/+^ mice was significantly increased compared with the AQP1^−/−^ mice following MI [Fig. 6(a)]. In order to confirm the above results, we analyzed the expression of the apoptosis proteins caspase3, bax and bcl-2 via immunoblotting. As expected, the expression levels of caspase3 and bcl-2 were significantly increased in the AQP1^+/+^ mice compared with the AQP1^−/−^ mice following MI, whereas bax expression was significantly decreased in the AQP1^+/+^ mice compared with the AQP1^−/−^ mice [Fig. 6(b)]. These results indicated that AQP1 deficiency protected against the development of cardiac dysfunction by attenuating cardiac apoptosis.

## Discussion

To date, cardiac AQPs have not been investigated to the degree that renal and brain AQPs have been. Previous studies have described multiple AQP expression patterns in the heart via RT-PCR and Western blotting^15^, and have also demonstrated that AQP1 and AQP4 are expressed in the mouse heart; however, the water permeable AQP4 has not been detected in the cardiac vesicles of mice. Controversy exists regarding the functional roles of AQPs^18^,^19^,^20^. Evidence pertaining to AQP1 expression in the heart is consistent with the data from this study. Our studies have demonstrated that AQP1 is highly expressed within endothelial cell membranes as opposed to myocardial cells, in mice. This pattern of AQP1 expression is an important determinant of its function. However, the physiologic, metabolic, and anatomic effects of AQP1 expression in cardiac tissue are not different under physiological conditions in both AQP1^+/+^ and AQP1^−/−^ mice.

Although previous studies have provided functional data indicative of the involvement of AQP1 in cardiac pathophysiology^12^, said data has not enabled researchers to elucidate the relationship between AQP1 expression and cardiac ischemia and infarction in mice. The findings of this study demonstrated the role played by AQP1 in the regulation of cardiac function following MI. Increased AQP1 expression levels were observed in cardiac tissue following the ligation of the LAD. AQP1 gene knockout in mice resulted in preserved LV function and reduced interstitial edema, as well as enhanced survival (data not shown). Infarct size is the most critical determinant of myocardial injury and mortality following MI^21^. The results of p-nitro-blue tetrazolium staining were characterized by reduced myocardial infarct size, and a serum analysis of myocardial enzyme levels (data not shown) demonstrated reduced cardiac injury following the ligation of the LAD in the AQP1^−/−^ mice. We also induced MI in AQP3^−/−^ mice in order to determine if AQP3 deficiency resulted in impaired kidney water absorption and decreased cardiac load^22^. We surmised that other AQPs may compensate for the absence of AQP1. Similar infarct sizes, myocardial enzyme levels and myocardial water content were observed in both the AQP3^+/+^ and the AQP3^−/−^ mice following MI. These findings support the hypothesis that AQP1 is associated with the pathophysiology of MI.

Edema is a generic component of the tissue response to acute injury. Myocardial ischemia, infarction and apoptosis may induce the development of myocardial edema, which contributes to cardiac dysfunction. The enhanced tissue edema development has been noted in several distinct AQPs such as AQP1^10^, AQP4^8^ and AQP5^9^ in many tissue types, suggesting that AQP-facilitated transmembrane water transport is responsible for the development of edema. Therefore, in the setting of conditions in which altered water homeostasis occurs, myocardial interstitial space enlargement occurs due to the efflux of fluid from the vascular compartment via AQP1, resulting in myocardial edema. Many studies have determined that small increases in myocardial water content are associated with significant ventricular systolic and diastolic dysfunction during the acute stage of MI^23^,^24^,^25^,^26^,^27^.

In the present study, we chose the time point of 18 h following the ligation of the LAD as the optimal time for the measurement of myocardial water content. Myocardial edema develops and peaks earlier than necrosis^28^. AQP1-dependent myocardial interstitial edema was reduced in the AQP1^−/−^ mice and correlated strongly with attenuations in myocardial injury but did not significantly ameliorate defective microvascular leakage following MI, with the exception of attenuating endothelial permeability. AQP1, a facilitator of endothelial cell water transport, represents the primary pathway by which the influx of O_2_ into epithelial cells the efflux of intracellular O_2_ to myocardial cells occurs^29^,^30^,^31^. However, AQP1-dependent myocardial edema increases the distance between coronary arterial supply and cardiomyocytes and prolongs oxygen diffusion in the myocardial interstitium, resulting in the exacerbation of myocardial ischemia, cellular apoptosis and worsening infarction^32^,^33^.

Additional key mechanisms underlying the development of severe cardiac injury in AQP1^+/+^ mice following MI may also involve the release of cytokines via AQP1 in endothelial cells in the setting of ischemia^17^. When the myocardium is deprived of blood secondary to coronary artery occlusion, ischemia, infarction, and myocardial remodeling are initiated. HIF-1α is one of the earliest responses to myocardial ischemia and infarction^16^.

During the early phase of MI, the level of HIF-1α expression was rapidly upregulated in the AQP1^+/+^ mice compared with the AQP1^−/−^ mice. The upregulation of AQP1 expression in endothelial cells affects not only water permeability but also O_2_ transport, which facilitates cytosolic O_2_ diffusion across the cell membrane^34^, resulting in the up-regulation of the expression of HIF-1α; however, AQP1 deficiency results in increased HIF-1α levels, which may contribute to cell death, tissue destruction, and the development of edema^35^. Additionally, recent study demonstrated that the genetic sequence of AQP1 is identical to the consensus sequence of the HIF-1α binding site^29^. Our results are indicative of a strong correlation between increased HIF-1α levels and the up-regulation of AQP1 expression, both of which may exacerbate the development of edema via increased microvascular endothelial permeability at 36 h following MI in AQP1^+/+^ mice. By contrast, AQP1 deficiency slightly increased both HIF-1α levels and myocardial edema, a finding that suggests that HIF-1α and AQP-1 are part of a single molecular cascade. Therefore, it is possible that HIF-1α may be induced by ischemia, resulting in increased microvascular permeability and AQP1-dependent myocardial edema.

In conclusion, we have demonstrated that AQP1 deficiency attenuates infarct size. Our results also suggest that the underlying mechanisms for this salutary effect may involve the inhibition of the development of AQP1-dependent myocardial edema, as well as decreased AQP1-dependent HIF-1α expression in endothelial cells, which decreases endothelium permeability and results in both decreased edema and decreased cellular apoptosis. Our results suggest that AQP1 inhibitors may represent a novel therapeutic target in the modulation of cardiac function in the setting of myocardial ischemia and injury.

## Materials and Methods

### Mice

AQP1^−/−^ and AQP3^−/−^ mice with a CD1 genetic background were generated via targeted gene disruption as described previously^36^. CD1 male mice and eight- to ten-week old and age-weight-matched AQP1^−/−^ and AQP3^−/−^ mice were used. The mice were maintained in a specific pathogen-free grade animal facility under a 12-h light-dark cycle. All procedures were approved by the Committee on Animal Research of Dalian Medical University and followed the ARRIVE guidelines pertaining to animal experimentation^37^.

### Induction of MI

MI was induced via permanent ligation of the left anterior descending (LAD) coronary artery in the AQP1^+/+^ and AQP1^−/−^ mice, as previously described, with modification^38^,^39^. Briefly, the mice were anesthetized via 2% isoflurane inhalation but not ventilated and were fixed on an animal operating table with a heating pad. Under sterile conditions, a left thoracotomy was performed through the 4^th^ left intercostal space in order to expose the heart, and a 6–0 sterile silk suture was passed rapidly under the LAD, just below the tip of the left auricle. The control subjects underwent a sham operation in which no ligation took place. The heart was subsequently replaced in the intra-thoracic space, followed by the manual evacuation of any air and the closure of both the overlying muscle and the skin. The mice were placed on a heating pad while recovering from the anesthesia. The chest cavity was open for only a few seconds, as the mice would have died in the absence of mechanical ventilation. The survival rate for each surgery group ranged from 80%–90%.

### Measurement of infarct size

After 48 hours, the mice were anesthetized with 2% isoflurane inhalation and received injections of 1% Evans blue dye (i.v.) in order to stain the ischemic area at risk (AR). The hearts were then arrested during diastole via the administration of 10% potassium chloride through the right carotid artery, before being removed and cut into 3 transverse sections as follows: apex, middle and root ring (3 mm in thickness). The perfused myocardium was stained using Evans blue solution, whereas the occluded vasculature remained uncolored. After removing the right ventricular wall, the AR and non-ischemic myocardium were separated by following a line of demarcation between the stained tissue and the unstained tissue. In order to distinguish between the ischemic and the infarcted tissue, the AR was cut into small pieces and incubated with p-nitro-blue tetrazolium (NBT, 0.5 mg/ml, 20 min at 37 °C, pH 7.4) to stain the viable myocardium blue-red. In the presence of intact dehydrogenase enzymes (normal myocardium), NBT forms a dark blue formazan; areas of necrosis lack dehydrogenase activity and therefore do not stain^40^. Both the AR and the infarct size were calculated and expressed as a percentage of the AR. The total and infarct areas of the left ventricle were measured using planimeter in a double-blinded manner.

### Assessment of hemodynamics

At 18 hours following MI, the mice were anesthetized via the inhalation of 2% isoflurane with ventilation; rectal temperatures were maintained between 36.7 and 37.3 °C. The left ventricle (LV) was catheterized using a conductance catheter connected to a pressure transducer (Chengdu Taimeng Technology Corp., LTD) via the right common carotid artery and carefully introduced into the LV in order to measure the heart rate (HR), systolic pressure (LVSP), and end-diastolic pressure (LVEDP), as well as the maximum and minimum rates of left ventricular pressure development (+d*P*/d*t*_max_ and −d*P*/d*t*_min_, respectively)^41^. LV pressure was recorded at steady state for 3–5 min using a 4 channel data acquisition system (Chengdu Taimeng Technology Corp., LTD).

### Measurement of myocardial water content (%MWC)

Myocardial water content was analyzed at both 18 and 36 hours following MI in the AQP1^+/+^ and AQP1^−/−^ mice as previously described^42^. In brief, the mice were anesthetized via the inhalation of 2% isoflurane, and the left ventricle was perfused with PBS. The hearts were subsequently arrested during diastole with 10% potassium chloride before being removed and dried with filter paper and weighed, before being dried again in a heat oven at 80 °C for 72 hours. The dried hearts were re-weighed, and the percentage of myocardial water content was calculated as [(wet weight-dry weight)/wet weight] × 100.

### Assay of microvascular permeability

The mice were injected intravenously with 10% fluorescein-isothiocyanate (FITC)-dextran (1.5 ml/kg; 40 kDa, Sigma, USA) 15 min before they were euthanized as previously described^43^. At 18 and 36 hours following MI, the mice were anesthetized via the inhalation of 2% isoflurane, and the left ventricle was perfused with PBS. The hearts were removed and homogenized before being centrifuged at 12,000 × *g* for 15 min at 4 °C. The fluorescent concentration in the supernatant was measured using a fluorescence spectrophotometer (an excitation wavelength 495 nm and an emission wavelength 517 nm). A standard curve was prepared using known amounts of FITC-dextran in order to calculate the concentrations in and the total amounts recovered from the cardiac tissues.

### Histopathology and immunostaining

The mice were anesthetized via the intraperitoneal injection of phenobarbital sodium (65 mg/kg) and perfused transcardially with 4% paraformaldehyde in PBS. The hearts were removed and fixed for 24 hours in 4% paraformaldehyde at 20 °C. The tissues were dehydrated using increasing concentrations of ethanol and embedded in paraffin. The sections were deparaffinized and stained using hematoxylin and eosin (H&E) in order to detect cardiac edema and morphological changes.

For AQP1 immunohistochemistry, the paraffin-embedded sections were cut at a thickness of 5 μm and deparaffinized before being treated with citrate buffer using microwave antigen retrieval and 3% hydrogen peroxide. The sections were incubated for 2 hours with a primary AQP1 antibody (1:100, Santa Cruz, CA, USA) before being incubated with a biotinylated secondary antibody (1:500, Vector Labs, Burlingame, CA, USA) and an avidin-biotin peroxidase complex (1:500, Vector Labs, Burlingame, CA, USA). Peroxidase labeling was visualized using diaminobenzidine (Vector Labs, Burlingame, CA, USA), which yielded a brown color.

In order to analyze the apoptotic cells, five-micrometer sections were prepared. The sections were subsequently stained using a fluorescein *in situ* cell death detection kit (Roche, Basel, Switzerland). Six images per heart (6 hearts per genotype group) were acquired. The number of TUNEL-positive cells per section was counted and calculated as a percentage of all nuclei according to the manufacturer’s protocol.

### RT-PCR and Western Blot Analysis

For RT-PCR, total RNA was isolated from mice via homogenization in TRIzol reagent (Invitrogen, Carlsbad, CA, USA). Following reverse transcription, PCR was completed using gene-specific primers designed to amplify the mouse AQP1 coding sequence. The primers were as follows: 5′-TGTATGCCTCTGGTCGTACC-3′ (sense) and 5′-CAGGTCCAGACGCAGGATG-3′ (antisense) for β-actin, and 5′-CTCCCTAGTCGACAATTCAC-3′ (sense) and 5′-ACAGTACCAGCTGCAGAGTG-3′ (antisense) for AQP1. Fluorescence-based real-time reverse transcription-PCR (RT-PCR), using 2 μg cDNA, was carried out using a LightCycler with FastStart DNA MasterPLUS SYBR Green I kit (Roche Diagnostics, Indianapolis, IN, USA). β-actin was used as a reference gene, and pooled cDNA from the AQP1^+/+^ mice, both before and after MI, was used as a calibrator. The results were reported as normalized and calibrated ratios.

For Western blotting, the mouse heart tissues were homogenized and centrifuged at 5,000 × *g* for 10 min. The supernatant was subjected to 10% SDS-polyacrylamide gel (20 μg of protein/lane) electrophoresis and transferred to a polyvinylidene difluoride membrane. The membrane was subsequently incubated with a primary antibody (1:100 or 1:500, Santa Cruz Biotechnology, Abcam, Cell signaling or Invitrogen, USA) overnight at 4 °C. Following washing with TBS, the membrane was incubated with a horseradish peroxidase (HRP)-linked secondary antibody at room temperature for 1 hour in the dark. The bands were developed using enhanced chemiluminescence (ECL; Thermo Scientific, Rockford, IL) and exposed on X-ray films (LIEGTLABS, Dallas, TX, USA). Band density was analyzed using ImageJ software.

### Statistical analysis

The data are presented as the mean ± standard error of the mean (SEM). All experiments were performed in triplicate. All data were estimated using Student’s *t*-test for two-group data sets and one-way ANOVA followed by Bonferroni’s post-hoc test to compare more than two groups, using GraphPad Prism, version 5.0 software. Statistical significance was determined as *P* < 0.05.

## Additional Information

**How to cite this article**: Li, L. *et al.* Aquaporin-1 Deficiency Protects Against Myocardial Infarction by Reducing Both Edema and Apoptosis in Mice. *Sci. Rep.* **5**, 13807; doi: 10.1038/srep13807 (2015).
